# Radiation-Resistant Er^3+^-Doped Superfluorescent Fiber Sources

**DOI:** 10.3390/s18072236

**Published:** 2018-07-11

**Authors:** Chengxiang Liu, Xu Wu, Jianhui Zhu, Nie He, Zhuoyan Li, Gongshen Zhang, Li Zhang, Shuangchen Ruan

**Affiliations:** 1 Guangdong Provincial Key Laboratory of Micro/Nano Optomechatronics Engineering, College of Mechatronics and Control Engineering, Shenzhen University, Shenzhen 518060, China; chxliu@szu.edu.cn; 2 College of Sino-German Intelligent Manufacturing, Shenzhen Technology University, Shenzhen 518118, China; wuxu@sztu.edu.cn (X.W.); 2160190401@email.szu.edu.cn (N.H.); 2160190403@email.szu.edu.cn (Z.L.); scruan@szu.edu.cn (S.R.);; 3 Shenzhen Key Laboratory of Laser Engineering, Guangdong Provincial Key Laboratory of Advanced Optical Precision Manufacturing Technology of Guangdong Higher Education Institutes, College of Optoelectronic Engineering, Shenzhen University, Shenzhen 518060, China; zhujianhui1@email.szu.edu.cn (J.Z.); 2161190230@email.szu.edu.cn (G.Z.); 4 College of Information Engineering, Shenzhen University, Shenzhen 518060, China; zhang_li@szu.edu.cn

**Keywords:** fiber optic sources and detectors, radiation, superfluorescence, radiation-resistant technique

## Abstract

The radiation effects of three Er^3+^-doped superfluorescent fiber sources (SFSs), which are based on three segments of Er-doped fibers with different lengths, are studied experimentally. We observed that the radiation-induced attenuation of the signal light of the 1530 nm band for an SFS is less than that of the 1560 nm band. Thus, the trimming technique of the Gauss-like spectra is investigated to reduce the mean wavelength drift. A filter was customized and used in superfluorescent fiber sources. To further reduce output power loss, the method with feedback control of pump power was adopted in the SFS. Then, the trimming spectral SFS with pump feedback control was tested under irradiation environment at the dose rate of 2.988 Gy/h. The experimental results demonstrate that the mean wavelength drift is <40 ppm and the loss of output power is <0.2 dB under a total dose higher than 1000 Gy. These findings confirm the significance of the method in improving radiation-resistant capabilities of fiber sources under irradiation environments.

## 1. Introduction

Interferometric fiber optic gyroscopes (IFOGs) have potential application in spacecraft and satellite space missions due to their numerous advantages, such as high reliability, long life, light weight, high resolution, as well as stable scale factor [1,2,3]. In meeting the requirements of navigation-grade IFOGs, its light source is usually an Er-doped superfluorescent fiber source (SFS) [1,2], whose high-stability mean wavelength and output power can achieve low bias drift and stable scale factor for IFOG application [3]. However, in space applications, various space rays and ionizing radiation can remarkably affect the radiation-induced attenuation (RIA) of SFSs, which in turn can cause mean wavelength drifts and power attenuation [2,3,4,5,6,7,8,9,10,11,12,13].

Most of the previous researches have been devoted to improving the radiation-resistant capabilities of SFSs [2,3,4,5,6,7,8,9,10,11,12,13]. The output power loss of SFSs can be mitigated by the Erbium-doped fiber (EDF) radiation-tolerant design and processing [13,14,15,16,17,18,19]. The loss can be further suppressed by the feedback control of the pump power [3,4]. On the other hand, the RIA-induced mean wavelength drift of an SFS can be effectively lowered by tailoring the spectrum [3,6,11,12,20], which is mainly achieved through filtering strategy, such as edge filter [3], band filter [6], and Gaussian filter [12,20]. Despite considerable research effort, the overall radiation-resistant performance of the SFS is far from state-of-the-art. At present, there are mainly three methods to improve the overall irradiation performance. Peng et al. [7,8,9] proposed reflectivity-tuning and green light photo-annealing methods to achieve a high-stability mean wavelength and keep the output power loss as low as 0.6 dB under a total dose higher than 2000 Gy. The band filter and pump wavelength tuning methods have been proposed by Liu et al. [6,10], which decrease the output power loss to 3 dB. Yang et al. [3,4] proposed the method of edge filter and closed-loop feedback control of pump current to manage the overall radiation-induced power attenuation and achieve a mean wavelength stability of 87.4 ppm.

However, the use of edge filters does not limit the mean wavelength drift well. Our previous work has shown that spectral trimming [11,12] and Er/Ce co-doped fiber [13] can effectively reduce the mean wavelength drift of the SFS and enhance the radiation tolerance of EDF. However, the output power loss could only be reduced to 2.616 dB under a total dose of 500 Gy. Consequently, an SFS with Gaussian spectral trimming and pump power feedback control technology is proposed in this paper. Under a total dose higher than 1000 Gy, the mean wavelength drift is less than 40 ppm and the output power loss is less than 0.2 dB.

## 2. Pre-Experiments Investigated

### 2.1. Radiation Experimental Setup

**Figure 1:**
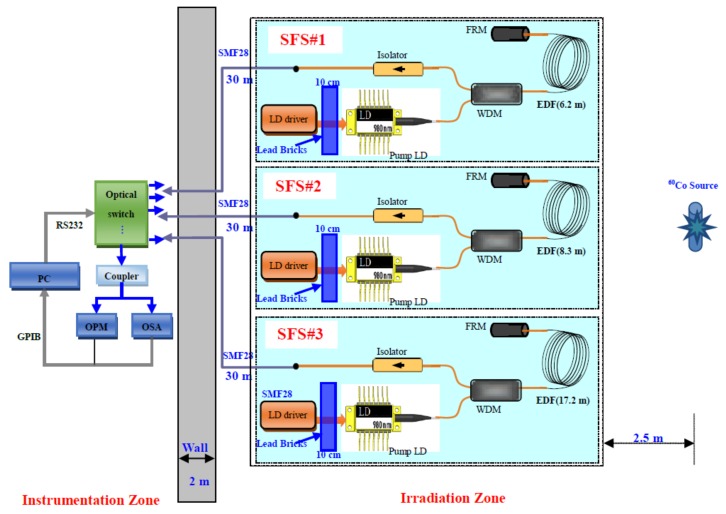
Experimental setup of SFS under Gamma irradiation.

An experimental setup was built (Figure 1) to investigate the radiation performance of SFSs. The experimental room was divided into two zones, namely, an instrumentation zone and an irradiation zone. The Gamma rays in the experiment were initially set to originate from a source with ^60^Co radiation. Irradiations were performed at room temperature under the dose rate of 2.988 Gy/h (i.e., total dose of approximately 500–1000 Gy). The dose rate (2.988 Gy/h) was set to be uniformly distributed around the irradiation source within a 2.5 m radius. The SFSs were placed in the irradiation zone. All optical components, such as EDF coils, pump laser diodes (LDs), wavelength division multiplexers (WDMs), Faraday rotator mirrors (FRMs), isolators, etc., were exposed to 2.988 Gy/h Gamma rays. However, the LD driver circuits were shielded with lead bricks. Then, at each end of the pigtail fibers of the SFSs, we spliced three pieces of 30 m SMF28 fibers (not submitted to irradiation) to propagate the output signal between irradiation and instrumentation zones. The measuring devices were placed in the nonirradiation zone. Finally, a mechanical optical switch (the end was connected to the coupler) was used to divide the signal light into two beams. The online output power of the SFSs was measured with an optical power meter (OPM). During the irradiation process, the changes in the spectral properties of the SFSs were recorded with an optical spectrum analyzer (OSA).

### 2.2. Tested Er^3+^-Doped Superfluorescent Fiber Sources

Three SFSs (SFS#1, SFS#2, and SFS#3) with double-pass backward (DPB) configurations were selected because of their high efficiency and stability performance [1,3,7] (Figure 1). The 980 nm pump light was introduced into the EDF through the WDM with 980/1550 nm. The pumped EDF produced amplified spontaneous emission (ASE) signals in the forward and backward directions. In the DPB configuration of the SFS, the backward ASE signals and the reflected forward ASE signals by an FRM were combined as outputs.

**Figure 2:**
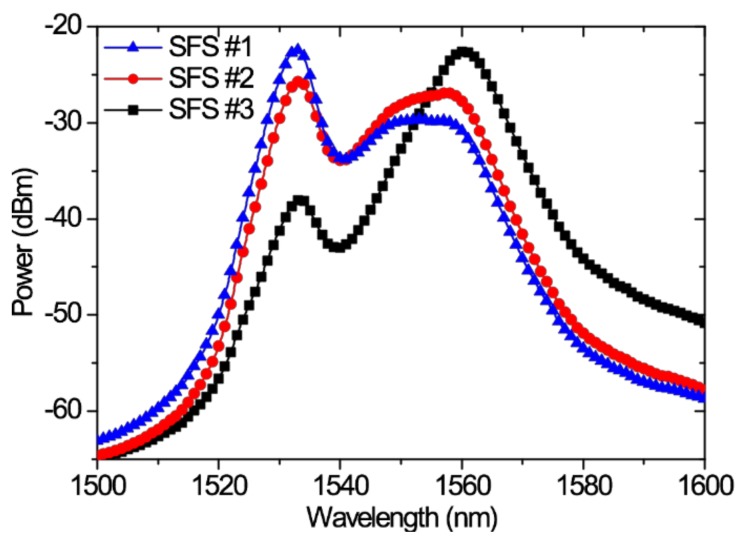
Output spectra of three SFSs with different EDF lengths at room temperature.

The abovementioned three SFSs were constructed with EDFs of different lengths (6.2, 8.3, and 17.2 m). These EDFs were then used as the gain media of the three SFSs. Consequently, the spectra varied because of the different EDF lengths. At room temperature (Figure 2), the different output spectra of the three SFSs were apparent at the bands of 1530 nm (SFS#1), 1530 and 1560 nm (two-peak equilibrium) (SFS#2), and 1560 nm (SFS#3). Table 1 lists the main characteristics of the three SFSs, in which only the EDF lengths are adjusted. When the drive current of the LD was 150 mA, the pump power output was approximately 70 mW.

### 2.3. Influence of Gamma Radiation on SFS Output

The three SFSs were constructed, and all of the parameters for the SFSs were the same except for EDF length. Before the radiation experiment, the stabilities of the SFSs were tested at room temperature for approximately 25 h. The results are shown in Table 2. The variations of mean wavelength and output power of the three SFSs are small. So, during the radiation experiment, the impact of temperature on the stabilities of the SFSs can be negligible.

**Figure 3:**
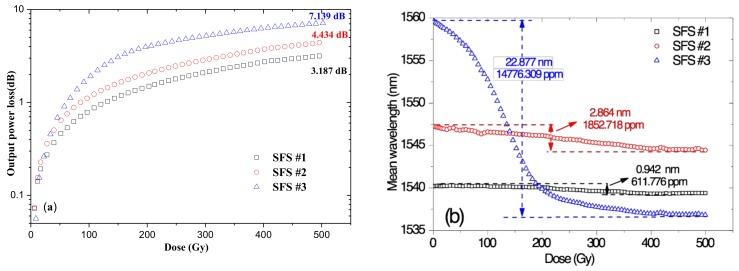
(**a**) Output power losses and (**b**) mean wavelength drifts of three SFSs with increased radiation dose.

Figure 3 shows the output power losses and the mean wavelength drifts of the three SFSs (SFS#1, SFS#2, and SFS#3). Lengthy EDFs can easily reduce radiation resistance performance; thus, the EDF should be chosen as short as possible. However, if the length of the EDF is too short, then the pump power conversion will be insufficient, which may lead to the very low output power of the SFS. In addition, in consideration of mean wavelength temperature stability (6.489 ppm, Table 2), the length of the EDF was set to 6.2 m. However, even if the mean wavelength shift (6.2 m) is smaller than 17.2 m, the value higher than 600 ppm cannot meet application requirements. In this study, other techniques were therefore considered to improve stability performance on the basis of mean wavelengths.

**Figure 4:**
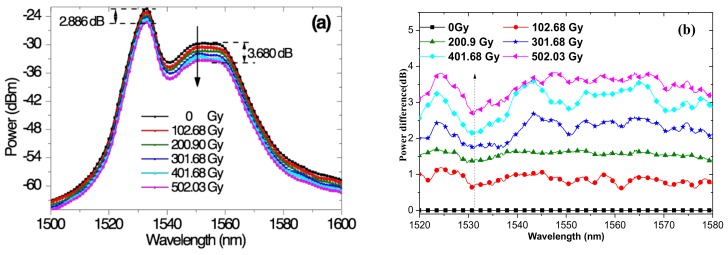
(**a**) Spectral evolution and (**b**) spectral variations of SFS#1 with respect to taking the zero-irradiated spectrum as the reference.

The spectral evolution of SFS#1 under Gamma radiation was investigated (Figure 4a). The spectra variations of the different doses were computed by taking the zero-irradiated spectrum as the reference (Figure 4b). In the figures, the bands near 1560 nm represent considerable loss while the spectrum near 1530 nm represent relative insensitivity to radiation. This indicates the suitability of the wavelength bands for space application, given that the spectrum near 1530 nm is always dominated by strong pump and strong gain. The result further suggests that spectral trimming is an effective method in resisting the radiation effect of SFSs.

## 3. Improvement of Radiation Resistance by Filtering Technology

### 3.1. Filter Design

**Figure 5:**
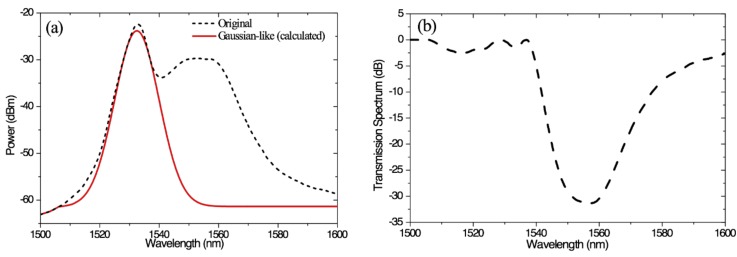
(**a**) Original spectrum and expected trimming spectrum and (**b**) transmission spectrum of the expected filter.

The effects of Gamma radiation on the SFS output near 1530 nm are relatively insensitive. Thus, we tailored the SFS spectrum at the left peak near 1530 nm to take the form of a Gaussian-shaped spectrum (i.e., see solid line in Figure 5a). The transmission spectrum of the corresponding filter is shown in Figure 5b.

### 3.2. Calculation of the Spectrum and Mean Wavelength of the Trimming SFS

**Figure 6:**
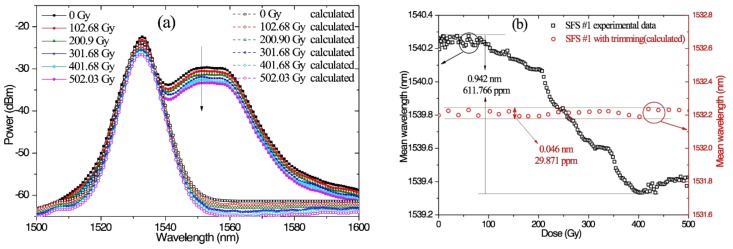
(**a**) Experiment results of the spectral evolution of SFS#1 and calculated Gaussian-like spectral evolution with irradiation dose and (**b**) mean wavelength drift of SFS#1 (experimental data) and calculated mean wavelength drift of SFS with trimming.

We calculated the SFS spectrum with the trimming (i.e., see Figure 4a) by using the designed filter, as shown in Figure 6. In Figure 6a, the evolution of the SFS spectrum with trimming almost overlaps. Then, the mean wavelength was computed as the weighted average of the signal wavelengths by using power spectral density as the weighting factor. The power spectrum *P(λ_i_)* at *n* equally spaced discrete wavelengths *λ_i_* can be expressed by the following summation [1],

$$\;\bar{\lambda }=\frac{\sum_{i=1}^{n}P\left(\lambda_{i}\right)\cdot \lambda_{i}}{\sum_{i=1}^{n}P\left(\lambda_{i}\right)}$$

The calculated mean wavelengths of the SFS#1 with trimming are shown in Figure 6b, which are obviously different from the experimental mean wavelengths of SFS#1. The mean wavelength drifts were negligible compared with SFS#1 without trimming. The calculated stability of the mean wavelength was low as 29.871 ppm, which suggests that the proposed method is theoretically feasible.

## 4. Experimental Results and Discussions

**Figure 7:**
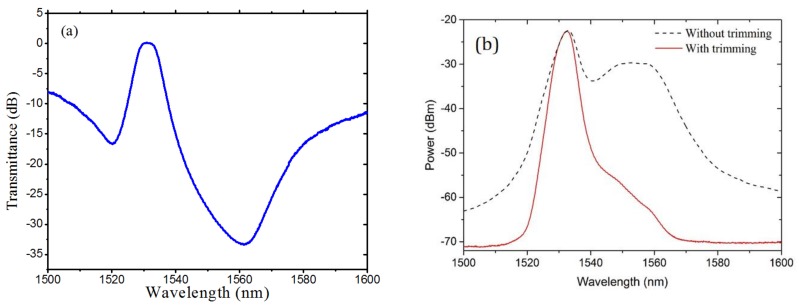
(**a**) Transmission spectrum of custom filter and (**b**) original spectrum and actual trimming spectrum.

On the basis of the above-mentioned filter design, the filter-based thin film was customized with pigtail fibers. The transmission spectrum is shown in Figure 7a. The bandwidth of the filtered spectrum (Figure 7b) is narrower than that of the ideal Gaussian spectrum (Figure 5a).

**Figure 8:**
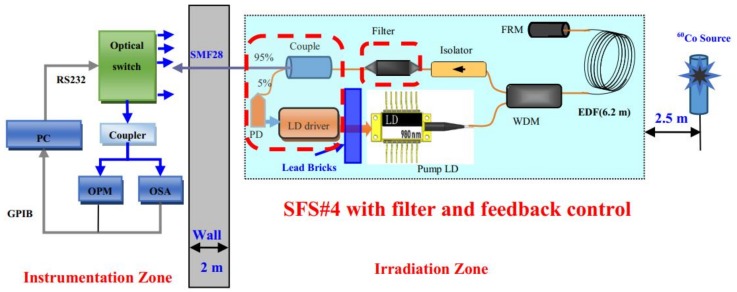
Schematic diagram of the feedback control SFS with the filter under Gamma irradiation. (This SFS is denoted by the symbol SFS#4).

Figure 8 presents the application of the filter and feedback control to the experimental setup (i.e., see Figure 1). The EDF length and the pump power used for the SFS were 6.2 m and 71 mW, respectively. The rest of the SFS components were exposed to rays, except the LD driver board that was shielded with lead bricks. The drive current of the pump LD was also adjusted by using the feedback control to compensate the radiation-induced power loss of the SFS.

**Figure 9:**
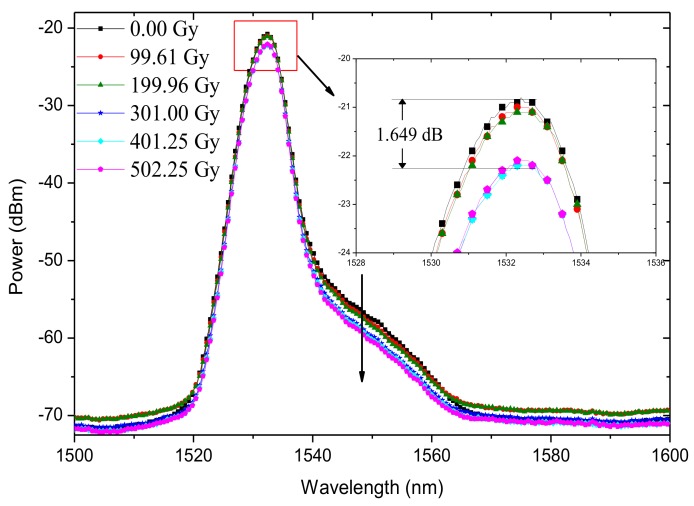
Spectral evolution of SFS#4 in increased radiation dose.

The output spectra of SFS#4 at different radiation doses are illustrated in Figure 9. The power spectrum of SFS#4 within the 1560 nm band, which was instability, was suppressed. In the case of DPB, a strong gain peak was observed near 1530 nm. Furthermore, the SFS output end was located in the strong pump end, which rendered the 1530 nm band emission to become more stable. Under the Gamma ray irradiation of 500 Gy, the spectral power of SFS#4 within the 1530 nm band exhibited attenuation of less than 1.65 dB, which can be regarded as being excellently stable.

**Figure 10:**
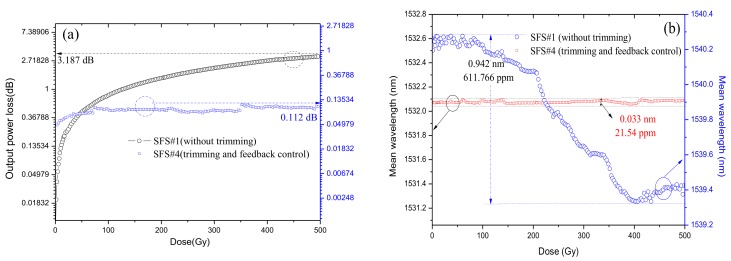
Experimental results of SFS#4 of (**a**) output power loss and (**b**) mean wavelength shift under Gamma ray irradiation of 500 Gy.

**Figure 11:**
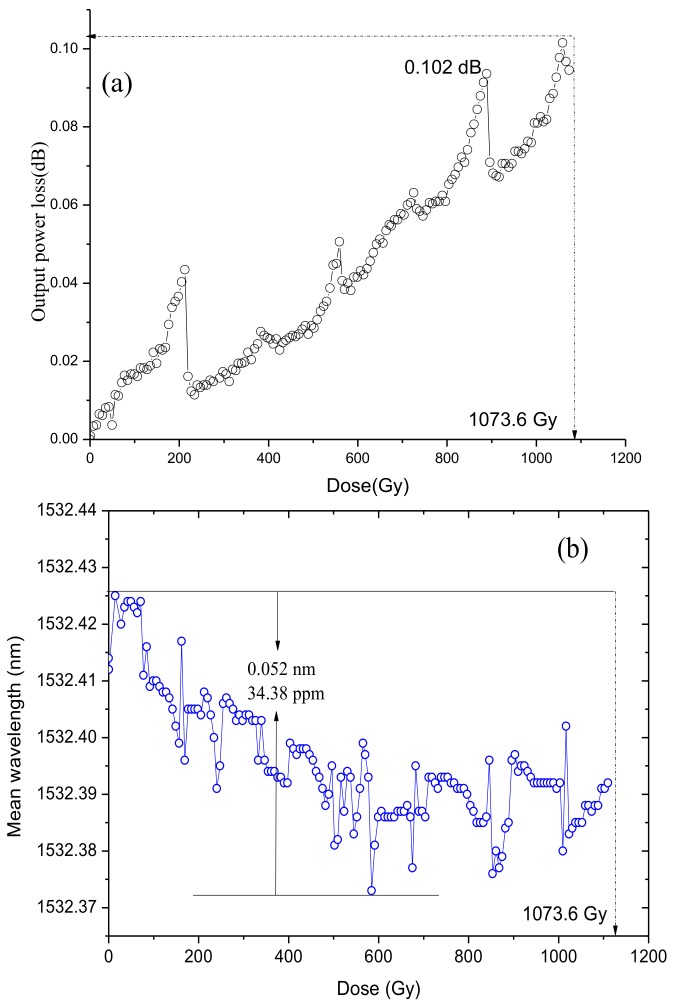
Measured (**a**) output power loss and (**b**) mean wavelength drift of an SFS with feedback control and trimming under Gamma ray irradiation of 1000 Gy.

Figure 10a compares the characteristics of the output power loss of SFS#4 to that of SFS#1. The result shows that the output power loss of SFS#1 can increase continuously in the entire range of the radiation dose. Under Gamma ray irradiation of 500 Gy, the output powers of SFS#4 and SFS#1 decreased by 0.112 dB and 3.187 dB, respectively. The mean wavelength drifts of two SFSs with increased radiation doses are shown in Figure 10b. The mean wavelength drift of SFS#4 attained a value of 0.033 nm (21.54 ppm). By contrast, the mean wavelength drift of SFS#1 (without trimming) sharply decreased with dose, and the value was 0.942 nm (611.766 ppm), which was approximately 28 times the value of SFS#4. To further confirm the capabilities of the radiation-resistant technique, another SFS with trimming and feedback control was tested under Gamma ray irradiation higher than 1000 Gy. The radiation-induced attenuation of the SFS was still insensitive to radiation, shown in Figure 11. The output power loss and the mean wavelength shift of the SFS were 0.102 dB and 0.052 nm (34.38 ppm), respectively.

## 5. Conclusions

We comprehensively investigated the radiation resistance performance of SFSs with spectral trimming technology. We demonstrated that an optimal filter can be used to reconfigure the spectrum to one with a Gaussian shape. Subsequently, the band spectrum (1530 nm in this study) was chosen mainly because it is in a strong pumping band, which makes the wavelength more stable. We designed a filter to take the form of a Gaussian-shaped spectrum. The SFS with trimming can achieve higher mean wavelength stability than the SFS without spectral trimming. In addition, the feedback control technology was used in the SFS, which can further reduce output power loss. The experiment results show that the mean wavelength drift of the SFS with feedback control and spectral trimming was less than 40 ppm, and the output power loss was less than 0.2 dB under a total dose higher than 1000 Gy. The obtained performance suggests that the proposed methods can effectively improve the radiation-resistant capabilities of SFSs in space environments.

## Figures and Tables

**Table 1:** Specifications of the Three Tested SFSs.

SFS	Configuration	EDF Length (m)	Pump LD Power (mW)	Output Power (mW)
SFS#1	DFB	6.20	70.00	14.96
SFS#2	DFB	8.30	70.00	15.90
SFS#3	DFB	17.20	71.00	18.59

**Table 2:** Stabilities of SFSs for 25 h at Room Temperature.

SFS	Mean Wavelength Shift (ppm)	Power Change (%)
SFS#1	6.489	0.402
SFS#2	11.627	0.472
SFS#3	11.539	0.658
